# Water on the Stove

**Published:** 1870-01

**Authors:** 


### WATER ON THE STOVE.

It is customary to put an open vessel of water on a stove, to prevent the air of a room in winter from getting too dry; thus, by filling the air with steam, it is rendered just as much less nutritious to the lungs and blood as if filled to that extent with the fumes from a dead carcass. This damp atmosphere has something of a steaming process on the skin, and thus renders the person extremely liable to take cold as soon as he goes into the open air. We want the air of a stove room ventilated, changed for pure air from without, not to fill it with the vapor of water. The only way yet known to improve the air of any room warmed by artificial heat, is to open a door or window from time to time, as while the family are at table eating their regular meals.

